# Myocardial micro-biopsy procedure for molecular characterization with increased precision and reduced trauma

**DOI:** 10.1038/s41598-020-64900-w

**Published:** 2020-05-15

**Authors:** Rikard Grankvist, Arvin Chireh, Mikael Sandell, Abdul Kadir Mukarram, Nasren Jaff, Ingrid Berggren, Hans Persson, Cecilia Linde, Fabian Arnberg, Johan Lundberg, Martin Ugander, Gioele La Manno, Stefan Jonsson, Carsten O. Daub, Staffan Holmin

**Affiliations:** 1 Department of Clinical Neuroscience, Karolinska Institutet, Solna, Sweden; 2 Department of Neuroradiology, Karolinska University Hospital, Solna, Sweden; 3 Department of Micro and Nanosystems, Royal Institute of Technology, Stockholm, Sweden; 4 Department of Biosciences and Nutrition, Karolinska Institutet, Solna, Sweden; 5 Department of Molecular Medicine and Surgery, Karolinska Institutet, Solna, Sweden; 6 Department of Clinical Sciences, Danderyd Hospital, Karolinska Institutet, Stockholm, Sweden; 7 Department of Medicine, Karolinska Institutet, Solna, Sweden; 8 Heart and Vascular Theme, Karolina University Hospital, Solna, Sweden; 9 Kolling Institute, Royal North Shore Hospital, and Northern Clinical School, Sydney Medical School, University of Sydney, Sydney, Australia; 10 Brain Mind Institute, School of Life Sciences, École Polytechnique Fédérale de Lausanne, Lausanne, Switzerland; 11 Department of Materials Science and Engineering, Royal Institute of Technology, Stockholm, Sweden; 12 MedTechLabs, Solna, Sweden; 13 Department of Clinical Physiology, Karolinska University Hospital and Karolinska Institutet, Solna, Sweden; 14 Charles Perkins Center, University of Sydney, Sydney, Australia

**Keywords:** Gene expression analysis, Interventional cardiology, Cardiovascular diseases, Heart failure

## Abstract

Endomyocardial biopsy is a valuable tool in cardiac diagnostics but is limited by low diagnostic yield and significant complication risks. Meanwhile, recent developments in transcriptomic and proteomic technologies promise a wealth of biological data from minimal tissue samples. To take advantage of the minimal tissue amount needed for molecular analyses, we have developed a sub-millimeter endovascular biopsy device, considerably smaller than current clinical equipment, and devised a low-input RNA-sequencing protocol for analyzing small tissue samples. In *in vivo* evaluation in swine, 81% of biopsy attempts (n = 157) were successful. High quality RNA-sequencing data was generated from 91% of the sequenced cardiac micro-biopsy samples (n = 32). Gene expression signatures of samples taken with the novel device were comparable with a conventional device. No major complications were detected either during procedures or during 7 days’ follow-up, despite acquiring a relatively large number of biopsies (median 30) in each animal. In conclusion, the novel device coupled with RNA-sequencing provides a feasible method to obtain molecular data from the myocardium. The method is less traumatic and has a higher flexibility compared to conventional methods, enabling safer and more targeted sampling from different parts of the myocardium.

## Introduction

Endomyocardial biopsy (EMB) is an established method for obtaining ventricular cardiac tissue for pathologic diagnosis and research, primarily for rejection monitoring after cardiac transplantation. EMB is also used in diagnosis of cardiomyopathies, infectious and neoplastic disease. Typically, the EMB device is inserted into the femoral vein or the right internal jugular vein and advanced to the right ventricle (RV), where samples are taken from the ventricular septum^1^.

The use of EMB is declining^2^, despite being the gold standard method for a number of diagnoses and being supported by cardiology organizations^1,3^. This decline may be caused by an increasing use of non-invasive low-risk tests, low diagnostic yield, and complication risks. In fact, the diagnostic yield of EMB is low for many diseases^4^. Moreover, the method has a significant complication risk, variably reported between 2.7% and 8.9%^1,2^.

We hypothesize that several of the shortcomings of EMB can be avoided with a significantly smaller and more steerable device. The current EMB devices are typically 1.66 mm (5F) or larger. Therefore, they can cause significant trauma at the site of biopsy and offer limited steerability within the ventricles. Consequently, few locations of the heart are regarded safe for sampling, with the right interventricular septum being the most common target^5^. Repeated biopsy sampling in the same area cause scarring and increasing difficulties to obtain fresh samples^5,6^. EMB also has a limited ability to detect diseases that reside within the left ventricle (LV), or in disease with patchy distribution of pathological changes^7^.

The aim of this study was to develop a “micro-biopsy” (micro-EMB) device that could be used in a micro-catheter. The device should have a different design of the cutting head to minimize endocardial trauma. A micro-EMB device would also be less traumatic on the heart valves, allowing safer use in both ventricles and enable better steerability.

With a micro-biopsy device comes the challenge of analyzing sub-milligram tissue samples. In conventional EMB, the tissue samples are typically analyzed with conventional histology^8^. For micro-EMB samples, these techniques would not be feasible. We hypothesized that modern molecular analyses, such as RNA-sequencing (RNA-seq), could be used as a replacement as they require much smaller sample volumes^9^. Gene expression signatures can be used to characterize cell and tissue types as well as disease states^10^. Efforts to find characteristic gene expression signatures for transplant rejection have already shown promising results in human kidney and heart biopsies^11–13^.

We present a new design of a micro-biopsy device, along with RNA isolation protocols and results from RNA-seq analysis and safety evaluation in *in vivo* porcine trials.

## Results

### Micro-bioptome design

**Figure 1:**
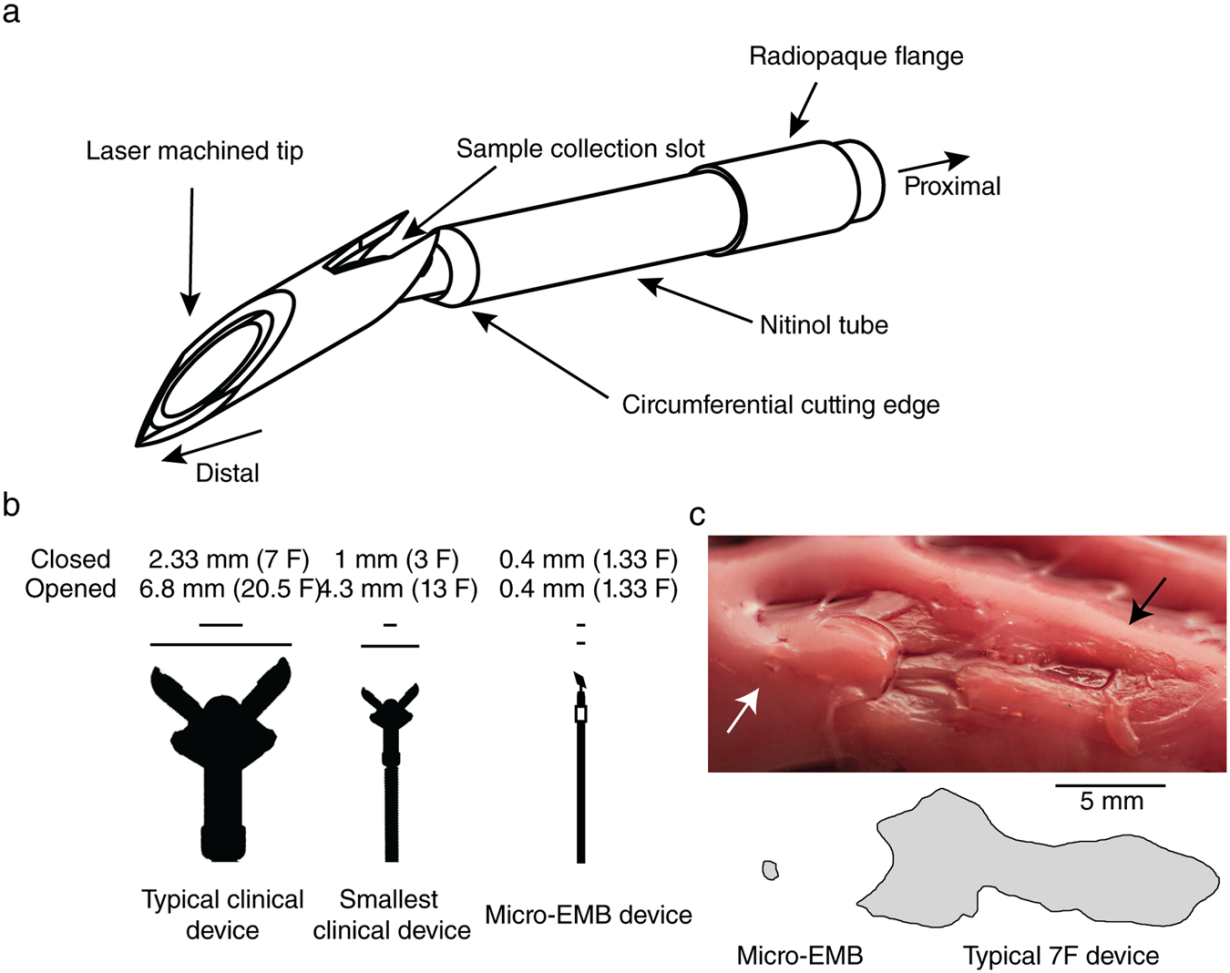
Micro-biopsy device design. (**a**) The micro-biopsy device features a movable cutting tip inside a cutting tube with a depth-limiting flange. The device is inserted into the cardiac ventricle wall until the collar abuts the wall (usually with a penetration depth of 2–3 mm). The tip is then advanced further into the tissue, rotated and retracted to cut and catch a sub-milligram piece of myocardial tissue. **(b)** Size comparison with clinically used devices. The micro-EMB device does not enlarge during sampling procedure, causing minimal trauma to the cardiac ventricle wall. **(C)** Ventricular wall surface after *ex-vivo* application of a conventional EMB device (black arrow) and the micro-EMB device (white arrow) on the left ventricle of swine. Affected regions are outlined below the image, and shows a reduced size of endocardial surface trauma from the micro-EMB device compared to the conventional EMB device (*n* = 3 replicates).

Through iterative testing and miniaturization, a device design small enough to fit inside a standard micro-catheter was achieved. The size and angles of the cutting head and the sample collecting slot, as well as penetration depth and the design of the cutting tube, was varied. The final design used an average penetration depth of 2.90 (SD 0.17) mm. Safety and efficiency evaluation of 65 device prototypes, by taking a total of 693 samples over 828 attempts in 23 swine (median 33 samples per animal) yielded in a final device design as depicted in Fig. 1a. A bi-directional cutting mechanism, consisting of two components, sits at the distal end of the device. The first component inserts into the ventricle wall and the mechanism allows for a cutting retraction against the second component, collecting a tissue sample at the first components’ proximal end. The device has an outer diameter (OD) of approximately 0.4 mm. and is housed in a 0.9 mm (2.7F) high-flow micro-catheter. The device is considerably smaller than currently available endomyocardial bioptomes (Fig. 1b). It caused a significantly smaller injury to the ventricle wall when taking a sample, compared to current clinical devices (Fig. 1c). The mean affected area was 0.266 mm^2^ (SD 0.155) with the micro-biopsy device, compared to 17.781 mm^2^ (SD 4.437) with the conventional device in an *ex vivo* application (Wilcoxon rank sum test, W = 30, p = 0.00433, *n* = 11).

### *In-vivo* sampling performance of selected design

**Figure 2:**
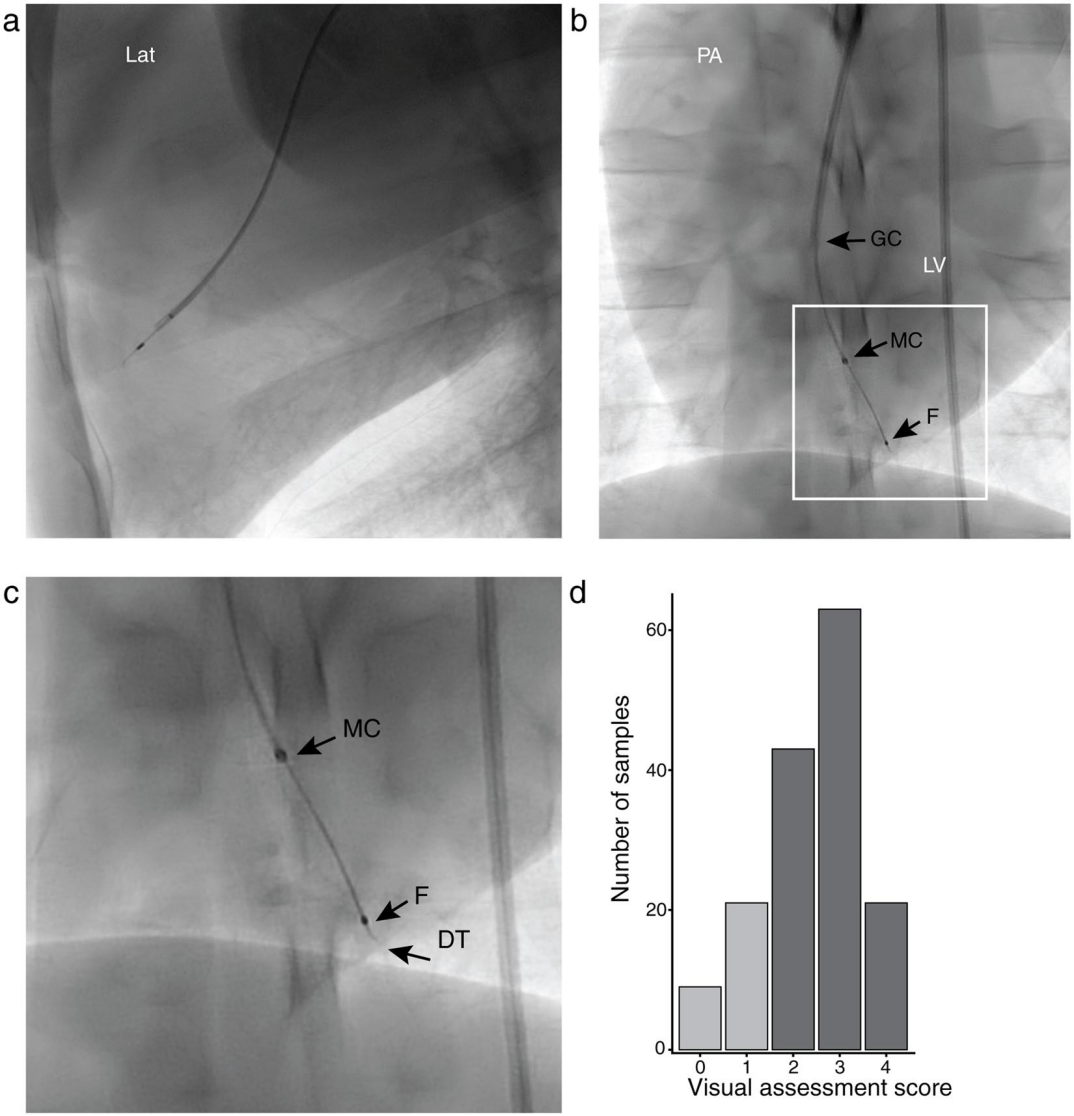
Fluoroscopy of the micro-EMB device inside the left ventricle (LV), assessed from lateral (Lat) and posterioanterior (PA) views. Various areas of the anterior, septal, posterior and lateral ventricle wall can be sampled (**a**,**b**). (**b**) is magnified in panel (**c**) where the guide catheter (GC), housing the micro-catheter (MC), the flange of the micro-bioptome (**F**), and the device tip (DT) is seen (*n* = 6 animals). **(d)** Distribution of scores for all biopsy attempts (*n* = 157, *n* = 6 animals). Scores were visually assessed by microscopy upon retraction, using a subjective scale ranging from 0 (no sample) to 4 (large protruding sample). There was no difference in RNA-seq quality or overall gene expression signature between samples with a score of 2–4 (dark grey). Samples with a score of 0 or 1 were considered as failures (light grey).

Due to its small OD and flexibility, the device could be steered to different parts of the ventricle wall (Fig. 2). To evaluate the ability to collect adequate samples, an initial visual assessment of the biopsy sample was made after each attempt. Based on the visual appearance, 127 out of 157 biopsy attempts (81%) were considered successful (Fig. 2d). The mean sample wet weight (measured on a subset of samples, *n* = 6) was 0.052 mg (SD 0.023).

### Tissue characterization by histology and RNA-sequencing

**Figure 3:**
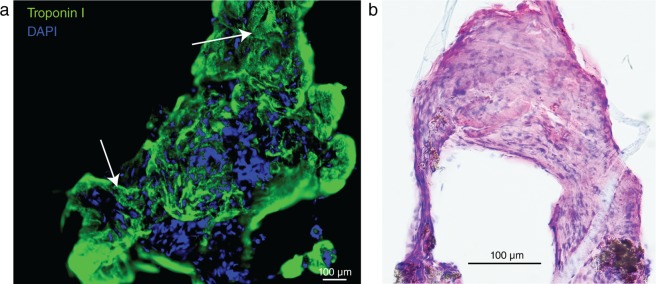
Histology of micro-EMB tissue (*n* = 3 animals). **(a)** Immunofluorescence of a micro-EMB sample procured *in vivo* in swine shows characteristic striation (white arrows) and ubiquitous positive troponin I antibody staining (green), confirming myocardial tissue. **(b)** Conventional hematoxylin & eosin stain of *in vivo* micro-EMB sample. The tissue samples are too small to effectively section, and will have considerable crush artifacts, making traditional histopathological evaluation difficult.

To verify that samples contained myocardial tissue, immunofluorescence was used to evaluate the presence of troponin I in a subset of samples (*n* = 7, Fig. 3a). As expected, the small tissue samples were difficult to section, limiting the use of conventional histology (Fig. 3b).

Due to the small size of the tissue samples, we adapted an existing RNA-seq protocol to accommodate the small input material. RNA-seq was performed on 54 samples of different types, including controls (Table 1, Supporting File S1). An initial quality control (QC) step was performed to exclude poor samples from further analyses. 29 out of 32 micro-EMB samples (91%) and all 22 control samples passed the QC. Three micro-EMB samples were excluded due to poor genome mapping rates and an overall low number of expressed genes. In the remaining 29 micro-EMB samples, the median number of reads was 15.8 million (range 6.1–25.1), with 73.6% of the reads mapped to the pig genome (range 59.2–83.8%). For the control samples, the number of sequenced reads was 13.5 million (range 3.9–22.1), with a median mapping rate of 78.5% (range 71.3–81.9%). The number of expressed genes was 11408 (range 6111–17076) in the micro-EMB samples and 11304 (range 6807–16309) in the control samples. Overall, 14% of samples showed signs of blood contamination and were therefore excluded (Supporting Fig. S3 and Data File S2). After exclusion based on RNA-seq quality (*n* = 3) and blood contamination (*n* = 7), a total of 44 samples were included and used in subsequent analyses. In total, 81% of micro-EMB samples were included.

**Table 1:** Descriptive statistics of biopsies taken and sequenced in the *in vivo* trials. Sequenced samples included micro-EMB and control samples from 5 individual pigs. Micro-EMB and conventional EMB samples were taken either at baseline (day 0) or follow-up (day 7). *One animal was lost to follow-up in the non-safety evaluation group.

Sample type	Timepoint	Animals (n)	Biopsies taken per animal (median)	Sequenced biopsies (n)	Sequenced animals (n)
Micro-EMB	Baseline	6	15	20	5
	Follow-up	5*	15	12	4*
Conventional EMB	Baseline	6	3	6	2
	Follow-up	5	3	4	2
Blood	Baseline	6	3	6	2
	Follow-up	5	3	0	—
Skeletal muscle	Baseline	6	3	6	2
	Follow-up	5	3	0	—

**Figure 4:**
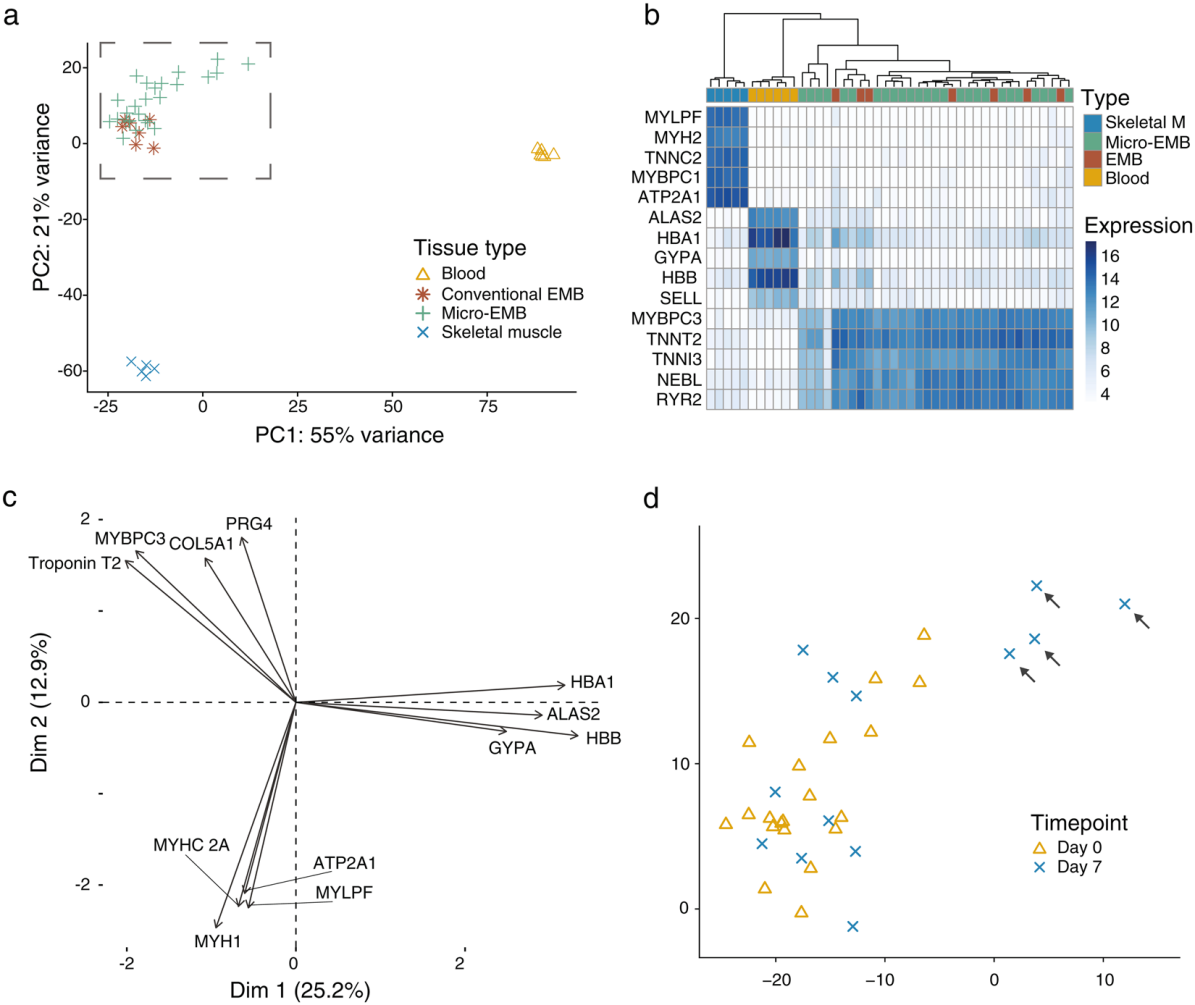
Transcriptomics of micro-EMB samples (*n* = 5 animals). (**a**) PCA plot of gene expression. Each point represents a sample (*n* = 44). Plotting of PC1 and PC2 shows separation of all three tissue types. Conventional EMB samples and micro-EMB samples cluster together, indicating high similarity. All genes apart from ribosomal RNA and mitochondrial genes were included (*n* = 25114). (**b)** Heatmap showing relative expression (normalized and transformed) of top 5 differentially expressed genes in each cluster. (**c**) PCA loadings plot shows top four weighing genes in PC1, PC2 and PC3, respectively. The genes are related to blood, cardiac muscle and skeletal muscle function. (**d)** Magnification of dashed rectangular area in (**a**). Four micro-EMB samples (black arrows) were markedly different from the other samples. Coloring and shaping based on timepoint for sampling shows that these four samples were all sampled on follow-up, indicating peri- or postprocedural cause. Gene expression comparison showed upregulation of genes related to “collagen fibril formation” and “wound healing”.

Gene expression comparisons between micro-EMB, conventional EMB and other tissue samples confirm that the novel miniaturized device does not alter the gene expression of the biopsy tissue. Principal component analysis distinctly separated the samples (*n* = 44) into three groups corresponding to tissue type (Fig. 4a). A heatmap of the top 5 differentially expressed genes for each tissue type shows homogenous expression within tissue types (Fig. 4b). The top loadings of the first three principal components (*PC1 – PC3*) were related to blood, skeletal muscle and cardiac muscle functions, respectively (Fig. 4c).

The micro-EMB samples showed higher variation compared to conventional EMB (Fig. 4d). To understand whether this was due to a technical or biological factor, the four outlier micro-EMB samples (as indicated in Fig. 4d) were compared with the micro-EMB samples with high similarity to the conventional EMB samples (*n* = 14). The four micro-EMB samples showed a higher expression of genes related to “collagen fibril organization” (adjusted p = 0.00001) and “wound healing” (adjusted p = 0.04), among other biological processes (Table 2, Supporting File S3). To look for signs of inflammation, we extracted the fold changes and corresponding p-values of 58 inflammatory genes as well as 22 macrophage related genes. Only 9 of 58 inflammatory genes and 1 of 22 macrophage related genes were upregulated (Supplemental files S4 and S5). All the four micro-EMB samples were obtained at follow-up, i.e. on day seven (Fig. 4d).

**Table 2:** Gene Ontology enrichment analysis of differentially expressed (DE) genes between outlier samples (*n* = 4) obtained at day 7 of follow-up and samples similar to conventional controls (*n* = 14). Intermediate samples, based on PCA plot appearance (Fig. 4d), were excluded to increase contrast in the comparison. The analysis shows enrichment of processes related to collagenization and response to injury, perhaps indicating that outliers represent resampling of a lesion created from a previous biopsy.

Biological process	Number of genes	Expected number of genes	Fold enrichment	P-value (adjusted)
Collagen fibril organization (GO:0030199)	12	0.93	12.88	0.00001
Response to hypoxia (GO:0001666)	17	3.23	5.26	0.0008
Wound healing (GO:0042060)	18	4.92	3.66	0.04

**Figure 5:**
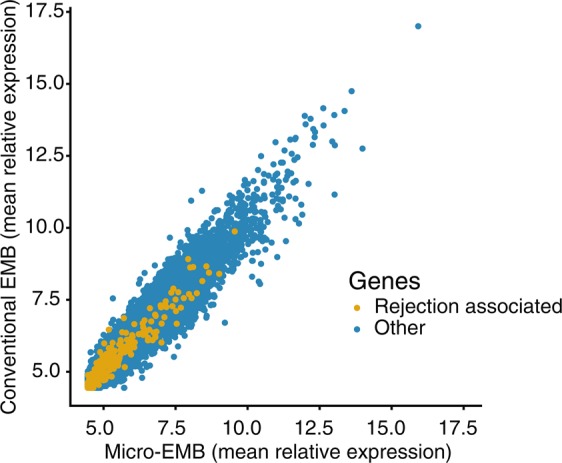
Relationship between relative expression (transformed and normalized) of all genes in micro-EMB and Conventional EMB group (*n* = 5 animals). Rejection associated genes are colored in yellow. The plot shows distinct linear correlation for all genes, including rejection associated genes.

Halloran *et al*. have previously identified 453 transcripts that were upregulated in rejection of human kidney and heart transplants^11^. From these 453 rejection-associated transcripts, we derived 301 ortholog pig genes. We tested visually whether these genes were similarly expressed between micro-EMB and conventional EMB samples. A scatterplot of average gene expression in both groups showed a similar relative expression between the groups (Fig. 5).

### Safety

During the development process of the device, various device iterations were evaluated in non-follow up *in vivo* experiments in 23 swine. One animal was sacrificed before the set end point of the experiment due to hypoxia. Necropsy revealed a pig bronchus configuration, causing bronchial endotracheal intubation. No animals suffered acute vascular or cardiac complications requiring treatment beyond the routine administration of amiodarone. In the follow-up safety study of the final device design, none of the animals (*n* = 3) had intra-operative complications during the procedures, apart from transient ventricular arrhythmias that did not require any intervention. All animals were successfully woken up and showed normal post-operative behavior. Transthoracic echocardiography at baseline and follow-up showed no measurable pericardial effusion, no aortic or mitral regurgitation and visually no abnormal regional myocardial thickening in any of the animals (Table 3). Left ventricular ejection fraction (LVEF) did not deteriorate between baseline and follow-up. On necropsy, all animals had small amounts (<10 ml) of blood-tinged pericardial effusion. There were small visible lesions on the ventricle wall, and sometimes surface of the heart, at the sites of biopsy attempts. There were no macroscopic signs of infarction or ventricular rupture on gross examination of the heart.

**Table 3:** Transthoracic echocardiography in the safety-evaluation group, performed on baseline (day zero) and follow-up (day seven). Left ventricular ejection fraction (LVEF) was not deteriorated. There was no overall thickening of the septal wall, measured in short axis view. There was no measurable pericardial effusion (Pex), aortic regurgitation (AR) or mitral regurgitation (MR).

Animal ID	Timepoint	LVEF (%)	Wall thickness (mm)	Pex	AR	MR
1	Baseline	59	8	0	0	0
	Follow-up	65	9	0	0	0
2	Baseline	66	10	0	0	0
	Follow-up	72	9	0	0	0
3	Baseline	55	8	0	0	0
	Follow-up	72	6	0	0	0

## Discussion

EMB analysed with classical histopathology remains the gold standard for diagnosis of several cardiac diseases, yet its use is being limited by low diagnostic yield and inherent risks with the procedure. Here, we present a novel biopsy device with a cutting portion of 0.4 mm, which is significantly smaller and more flexible compared to present biopsy devices measuring 6.8 mm (a 7 F device in the opened position, Fig. 1b). The reduced size of the device makes it less traumatic to the myocardium, endocardium as well as heart valves, and enables greater steerability. By design, the tip of the device enters the true myocardium like a needle before the tissue sample is collected. This design feature avoids the tearing of a larger tissue including endocardium, as in conventional EMB (see Fig. 1c). The 0.9 mm (2.7F) micro-catheter housing our device, coupled with an angled 1.33 mm (4F) guide catheter, enables targeted sampling from large parts of the myocardium of both RV and LV. Overall, these features of micro-EMB can potentially increase diagnostic yield as well as reduce complication risks, particularly in the case of repeated sampling such as in transplant monitoring.

Due to the reduced dimensions of the device, one aim was to test the consistency and quality of the resulting molecular data. Our results show that a majority of the biopsy attempts (81%) were successful based on the initial visual assessment. Of the sequenced micro-EMB samples in this study, the vast majority yielded high quality RNA-seq data (91%). Apart from sequencing quality, blood contamination affected the gene expression signatures of both some micro-EMB samples and controls, which were excluded. For future applications, measures can be taken to reduce blood contamination at time of intervention, such as washing the sample in saline or a red blood cell lysing buffer prior to snap freezing. Alternatively, bioinformatic methods can be used to correct for the blood contamination. The latter was not attempted in this study, as it could have compromised the interpretability of the results. In the clinical setting, multiple sample replicates are advisable for reliable histological diagnosis and molecular biology analysis. Typically, 5-10 samples are recommended for conventional EMB^1^. Our results suggest a smaller number of samples for the developed micro-EMB which, in addition, is significantly less traumatic. Conversely, since the trauma of each biopsy is smaller, the total number of biopsies taken in a session could be increased with micro-EMB, to attempt a greater diagnostic yield by sampling a larger area of the ventricular myocardium.

The gene expression comparison confirmed that the micro-EMB device reliably samples myocardial tissue. The reduced dimensions of the micro-EMB device did not seem to generally influence the gene expression signatures, as compared to conventional EMB samples. However, there was a larger variation within the micro-EMB samples. The 4 separated micro-EMB samples, all obtained at the follow-up sampling on day 7, showed upregulation of genes related to collagenization and wound healing. Most likely, these samples were taken from a region of focal injury caused by repeated sampling from the same region (a situation that could be avoided when using the micro-EMB device since it is easily steerable). An alternative explanation to the increased expression of collagen-related genes could be erroneous sampling of endocardium, particularly since there were few signs of inflammation in these samples. Regardless of the cause, this finding emphasizes the necessity of varying the sampling location in a clinical scenario as well as obtaining a correct penetration depth. In both these aspects, the micro-EMB device provide useful advantages compared to a conventional EMB device. For instance, in a clinical scenario where fibrosis is anticipated, the penetration depth could be increased to ensure that the device samples deep myocardial wall tissue, rather than endocardial fibrosis. This variable sampling depth is a unique feature of the device.

Efforts have already been made to deploy transcriptomic analyses on conventional EMB specimens for diagnosis of transplant rejection^11–13^. To give an indication of diagnostic performance of our micro-EMB procedure, we examined whether disease relevant genes were similarly expressed in conventional EMB and micro-EMB samples. The results showed that the average expression of all genes was highly concordant between the two groups, including the rejection associated genes (Fig. 5). This finding indicates that the reduction of sample size does not affect the relative expression of disease relevant genes, and that the diagnostic capabilities are retained despite the size reduction. However, classical histopathology still remains the gold standard for diagnosis of most cardiac diseases and transplant rejection. The usefulness of micro-EMB depends on the concurrent development of molecular diagnostic methods such as transcriptomics, proteomics or other methods that do not require large tissue samples. Important work has already been done in this field regarding transplant rejection, for instance with the development of the MolecularMicroscope^14^, as well as recent explorations of the role of microRNAs in rejection^15–17^.

Our 7-day follow-up safety group (*n* = 3), suggests that the method is safe, despite a relatively high number of biopsies per animal and session. The small OD of the device, as well as design features to limit tissue penetration depth, contribute to the safety. The pulling mechanism of the device secures the tissue sample upon retraction, preventing embolization. The complication risk from tissue trauma is most likely much lower in micro-EMB compared to conventional EMB, since the cutting portion of the device is significantly smaller. As shown, the endocardial lesion after a micro-EMB is smaller than from conventional EMB (Fig. 1c), which should reduce the risk for thrombocyte aggregation, thrombus formation, and possible embolization to the brain. As the dimensions are reduced, the risk of damaging heart valves should also be reduced, since smaller and more flexible guide catheters can be used to house the device. We found no mitral or aortic regurgitation after micro-EMB, a common indicator of valve injury.

This study was mainly performed to evaluate the technical and analytical feasibility, as well as safety, of the novel method. Consequently, the ability to diagnose transplant rejection or other disease states was not directly studied. Other authors are investigating the capabilities of transcriptomic analysis on heart muscle tissue^12,18^. It is still not known what role heart tissue based (in contrast to blood based) RNA-seq analysis will play in future diagnostics. In transplant monitoring, there will most likely be a need of heart tissue based diagnostics, despite recent attempts to reduce the need of invasive heart biopsies by the use of blood based tests such as AlloMap or cell-free DNA^19,20^. In this context, the novel micro-biopsy device may play an important role as a less invasive alternative to conventional EMB, which was developed decades ago with histopathological analyses in mind. Outside the heart transplantation field, direct myocardial tissue analysis may be of great interest for research and diagnostics in other circumstances such as unexplained heart failure, where currently EMB has a poor risk-benefit ratio. Apart from reducing the risks, the novel device might also increase the diagnostic yield due to the superior flexibility and steerability of the device. For instance, imaging guidance could be used with the micro-EMB procedure for targeting patchy cardiac disease such as in amyloidosis, myocarditis and transplant rejection^21–25^. Conversely, there will probably be some cardiac diseases for which molecular analyses will be unable to detect disease. In these cases, histology may remain the preferred method, and therefore disqualifying the use of a micro-EMB device. For other cardiac disease states, improvements in peripheral blood molecular analyses (“liquid biopsy”) or non-invasive imaging may also make tissue based diagnostic methods redundant. Therefore, we believe micro-EMB will not have a role in scenarios such as ischemic heart disease, uncomplicated heart failure or long-term follow-up of low risk heart transplant patients.

As in all animal studies, the difficulties in clinical translation must be considered. There are known differences between human and swine in their cardiac conduction systems^26^ and hemostasis^27^. There could also be differences in tolerance to myocardial provocation as well as other unknown factors relevant for safety evaluation. The follow-up safety trial featured relatively few animals, despite a large total number of biopsies. However, the device development process featured a larger number of animals (*n* = 23), and while the final device design was not studied in all of them, the development iterations were all similar with respect to overall size and mechanism of tissue penetration, indicating the safety of the method.

In conclusion, this novel technique for endomyocardial biopsy coupled to RNA-seq of small myocardial samples, is an important step towards less invasive access to myocardial gene expression, applicable to transplant monitoring and a wide variety of cardiac diseases. The micro-biopsy device could potentially replace current biopsy devices, to offer increased diagnostic yield and accuracy. Compared to conventional methods, the technique is less traumatic and may have a lower risk of complications, while allowing greater steerability to reach specific targets in both the right and left side of the heart.

## Methods

### Experimental design

The purpose of this study was to design a smaller, more steerable, and less traumatic endomyocardial biopsy (micro-EMB) device able to consistently sample tissue of sufficient quality for RNA-sequencing analysis. The study was divided into two main parts: development and optimization of the method; and evaluation of the method in an *in vivo* follow-up series in swine (Supporting Fig. S1).

In the first part, the device prototypes (*n* = 65) were designed, constructed, and evaluated in-house, either in an *ex-vivo* simulator or in acute non-survival pig experiments (*n* = 23). The samples that were collected in the *in vivo* trials were used to test and optimize RNA isolation protocols.

Then, a longitudinal study of six animals was performed to evaluate device performance, sample quality and safety. The animals were studied at day 0 and day 7. Animals were enrolled consecutively and were allocated to experimental groups before arrival to the animal housing facility and before the first intervention in each animal. The animals were divided into two groups, prioritizing sampling performance and safety measurements, respectively. The number of animals (*n* = 6) was selected to reach three individuals in each group. For the first three animals, a variety of sample types were collected, including conventional heart biopsies. For the following three, sampling was performed only by taking micro-biopsies to prioritize safety evaluation. RNA was sequenced on a subset of samples (*n* = 32), selected to represent micro-biopsies of all sizes from both timepoints (see Supplemental Data File S1 for complete description). Peripheral blood and skeletal muscle samples from the groin were sequenced for use as contrast in subsequent analyses. Primary safety endpoints were prospectively selected as death or signs of severe illness. Secondary endpoints included symptoms of cardiac disease or complications, including decreased left ventricular ejection fraction, tamponade and significant pericardial effusion, myocardial infarction, and arrhythmias.

### Ethical considerations

All research was conducted in accordance with national and local guidelines for Sweden and Karolinska Institutet respectively. All animal experiments had ethical approval from the local ethics committee (Stockholms Norra Djurförsöksetiska Nämnd, Stockholm, Sweden) and were performed in accordance with ‘Principles of Laboratory Animal Care’ formulated by the National Society for Medical Research and the ‘Guide for the Care and Use of Laboratory Animals’ prepared by the Institute of Laboratory Animal Resources and published by the National Institutes of Health.

### Micro-biopsy device prototyping and *ex vivo* evaluation

The device design was iteratively developed and manufactured in house. Different prototypes were iteratively designed, prototyped and tested *ex vivo* and *in vivo* to arrive at a final design with a cutting portion OD of 0.4 mm. The design features a movable bidirectionally cutting tip attached to a wire housed inside an OD 0.4 mm, circumferentially symmetric, cutting tube with a penetration depth-limiting radiologically visible flange. The tip extends beyond the cutting tube and can be advanced a limited distance coaxially in relation to the tube. The device penetration depth can be varied by altering the position of the radiopaque flange during manufacture. The average penetration depth used for the safety study was measured on a subset of devices (*n* = 9). The device is inserted into the ventricle wall until the flange abuts the wall. The tip is then advanced further into the tissue, rotated and retracted to cut and catch a sub-milligram piece of myocardial tissue. Fully advanced, the device tip will penetrate 1 mm into the ventricle wall beyond the initial penetration depth. A groove in the wire behind the cutting tip serves as a cradle for the harvested tissue, as it is withdrawn mostly into the tube, wedging to the cutting edge of the tube. The circumferentially symmetric cutting edge of the tube makes any orientation of the movable cutting tip possible while still obtaining samples. The device can then be removed without losing the tissue piece or contaminating it. *Ex vivo* evaluation was performed using direct puncture of post-mortem myocardium using the micro-EMB device. Tissue yield was evaluated with microscopy directly after extraction. Selected samples were inspected with basic histology. Some prototypes were tested in a custom-made simulator constructed from polyethylene tubing and an acrylic glass chamber, simulating blood vessel- and cardiac ventricle anatomy.

### *In vivo* evaluation

The most promising device prototypes were tested in non-follow up animal experiments, in a total of 23 naïve, adult, mixed-gender, Yorkshire-Swedish farm swine, weighing 37 (29–48.5) kg. Animals were pre-medicated with sedatives (intramuscular injection of tiletamin 2.5 mg/kg, zolazepam 2.5 mg/kg, medetomidin 0.1 mg/kg) and taken to a fully-equipped clinical angiography suite, intubated and mechanically ventilated while receiving standard surgical anesthetic care. Anesthestia was induced with i.v. propofol (20 mg) or i.v. sodium pentobarbital (120–180 mg) and maintained with sodium pentobarbital infusion (15–20 mg/kg/hour) or inhaled isoflurane (0.5–1.5%). Analgesia was achieved using i.v. fentanyl (100 µg/hour). Throughout intervention, the animals were monitored for complications using ECG, invasive blood pressure measurement, oxygen saturation, temperature, and urine production. Animals were treated with 75 mg amiodarone i.v., and an additional dose of 75 mg if the duration of the experiment surpassed 3 hours.

Early device prototypes were tested using transjugular access to the right ventricle using a 2.83 mm (8.5 F) deflectable sheath (Agilis NXT, Saint Jude Medical, Saint Paul, MI, USA). As prototypes were improved and miniaturized, devices were tested in both ventricles with access from the femoral artery and vein, respectively, and eventually only in the left ventricle (LV). All left ventricular biopsies were obtained using transfemoral approach. A 5F or 7F short sheath was used to gain access to the femoral artery, and a standard 1.66 mm (5F) or 1.33 mm (4F) straight diagnostic catheter (Torcon NB advantage, Cook Medical, USA) was used to access the LV.

The micro-biopsy samples were taken by advancing a 2.7F micro-catheter housing the biopsy device into the LV. In the LV, the micro-biopsy device alone was advanced into the myocardium. Inside the myocardium, the cutting tip of the device was advanced, rotated 180 degrees, and retracted to pinch off a tissue sample. The device was withdrawn together with the microcatheter to obtain the tissue. Conventional EMB samples were obtained using a standard endomyocardial forceps bioptome (3F or 5.2F Flexible Myocardial Biopsy Forceps, Cook Medical, USA or 6F Jawz Endomyocardial Biopsy Forceps, Argon Medical Devices, Frisco, TX, USA).

After retraction of the biopsy device, the samples were retrieved from the device by a single operator, with aid of a surgical microscope. The samples were graded by the operator on a semi-quantitative scale in the range 0–4, based on tissue size, appearance, and ease-of-handling. In short, complete lack of tissue in the device was designated as 0, and a large tissue protruding from the sampling pocket was designated as 4. The samples were stored in 0.2 ml PCR tubes and immediately put on dry ice.

At the end of experiment, animals were sacrificed using a lethal dose of sodium pentobarbital (100 mg/kg).

### Longitudinal evaluation of final device design

In 6 naïve adult female Yorkshire-Swedish farm swine, weighing 28.75 (27–31.9) kg, the micro-EMB device was evaluated during a 7-day follow-up period. Anesthesia and euthanasia was performed as described above. Micro-biopsy was either used as only intervention (*n* = 3) or in conjunction with conventional EMB (*n* = 3). Before and during follow-up, animals were housed in a dedicated large-animal housing facility with trained staff, with veterinarian supervision. The initial procedure (day 0) was performed as in the non-survival experiments described above, with the exception of using inhalation anesthetic (isoflurane) instead of sodium pentobarbital. Moreover, in addition to the intraoperative monitoring, transthoracic echocardiography was performed before starting the procedure on day 0 and day 7. Echocardiograms were performed using a VIVID E95 ultrasound machine with a M5S probe (GE Healthcare). The left ventricle was visualized from a left parasternal imaging window, in long axis and at mid ventricular level in short axis. Left ventricular ejection fraction (LVEF) was calculated by Teichholz’s formula. The indicated LVEF calculations are averages from three measurements. The aortic and mitral valves were visualized in parasternal and subcostal views.

The intervention consisted of 15 (12–17) micro-biopsies and three conventional biopsies (in the non-safety group only). After intervention, the animals were awoken from anesthesia and housed for seven days, receiving standard post-operative care, and monitored for complications. At day seven, a follow-up intervention was performed with 15 additional micro-biopsies (10 – 15) and three conventional biopsies (non-safety group only). At the end of day seven, the animals were sacrificed and the heart examined. A subset of samples was sequenced using RNA-seq. The subset of samples was selected in a way to achieve a mixture of sample scores (Supplementary file S1).

### Histological evaluation

Micro-biopsy samples obtained in the *in vivo* evaluation study were too small to reliably section onto slides, and had significant crush artifacts, making evaluation difficult. Instead, tissue pieces were placed directly on to microscope slides and compressed in a manner similar to the preparation of whole blood slides. Slides were stained using May-Grünwald-Giemsa, hematoxylin & eosin or immunfluorescence for troponin I using a mouse anti-swine primary antibody (ab19615, Abcam, Cambridge, UK) diluted 1:200 and donkey anti-mouse Alexa Fluor 488 secondary antibody (Invitrogen, Carlsbad, Germany) diluted 1:500, mounted with a DAPI counterstain (Invitrogen).

### RNA isolation and sequencing

RNA was isolated and sequenced from 54 samples of various tissue types and sizes (supplementary file S1). The samples were selected from five individual pigs in the longitudinal study. All samples were stored and processed in the same manner. For total RNA isolation, a modified version of Agencourt RNAdvance tissue kit (Beckman Coulter, Indianapolis, IN, USA) was used. The kit protocol was modified by reducing all reagent volumes to 1/13, due to the small sample sizes. In the homogenization step, the samples were vortexed and centrifuged with standard benchtop equipment. Any remaining bulk tissue, after homogenization, was discarded before proceeding with the protocol. The RNA isolation was performed in 11 different batches, in order to minimize bench-time for each sample. The samples were randomly assigned to each batch, using a random number generator in Microsoft Excel. After generation of gene expression data, as described below, the PCA plots were colored and shaped based on batch number to confirm that the expression patterns were not driven by batch effects. Libraries were prepared with the SMARTer Pico Stranded Total RNA-Seq Kit (Takara Bio Inc.), pooled and sequenced on the Illumina HiSeq platform (Illumina, San Diego, CA, USA) using 2×50 base pair reads.

### Statistics

Raw sequencing reads were trimmed with TrimGalore^28^, before mapping to the pig genome assembly Sscrofa10.2, using STAR RNA-seq aligner^29^. FeatureCounts^30^ was used to assign the reads to genes. All downstream analyses, starting from the gene count matrix, were performed using the R statistical computing language, version 3.4.1^31^. The number of genes expressed in each sample was calculated by converting the raw counts to counts per million (cpm), and calculating the number of genes that had a cpm value of 1 or higher. Gene counts were transformed and normalized with DESeq. 2^32^, using default settings. Upregulated genes in blood were selected with the following thresholds: log_2_ fold change > 1, adjusted p-value <0.1 and a base mean value > 10.

To examine the variation between the micro-EMB samples, the four micro-EMB samples indicated in Fig. 4d were compared with the micro-EMB samples that were similar to conventional EMB-samples (*n* = 14). Differential expression (DE) analysis was performed. Genes upregulated in the deviating micro-EMB samples were selected by using log_2_ fold change > 1, adjusted p-value <0.05 as threshold. The upregulated genes (*n* = 717) were tested for enrichment of biological processes by using the Gene ontology (GO) web interface using default parameters^33–35^. Bonferroni correction (default) was used to adjust p-values. To look for inflammatory and macrophage related genes more specifically, we obtained lists of characteristic genes from the literature^36,37^. We translated these genes to pig orthologs (Supplemental files S4 and S5) using Ensembl Biomart^38^. For these genes, we extracted the fold changes and associated statistics from the same DE analysis as already described.

To evaluate whether known disease relevant genes were captured by our method, we extracted allograft rejection associated transcripts (RATs) from the literature^11^. These transcripts were converted to swine, yielding 301 orthologs. The average relative expression of these genes, along with all other genes, was calculated and visualized (Fig. 5).

Descriptive statistics are presented as *median (range)* unless otherwise specified.

## Supplementary information

Supplementary information.

Supplementary information 2.

Supplementary information 3.

Supplementary information 4.

Supplementary information 5.

Supplementary information 6.

Supplementary information 8.

## Data Availability

All data associated with this study are available in the main text or the supplementary materials. The gene expression data have been deposited in NCBI’s Gene Expression Omnibus^39^, and are accessible through GEO Series accession number GSE124783 (https://www.ncbi.nlm.nih.gov/geo/query/acc.cgi?acc=GSE124783).
