# Supplementary material for: Myocardial micro-biopsy procedure for molecular characterization with increased precision and reduced trauma
Source: Sci Rep. 2020 May 15;10:8029. doi: 10.1038/s41598-020-64900-w (PMC7229024; doi:10.1038/s41598-020-64900-w)
Supplement: Supplementary file 6 — Supplementary information 6. [file 41598_2020_64900_MOESM6_ESM.pdf]

Myocardial micro-biopsy procedure for molecular characterization with increased precision and reduced trauma

Supplemental Data

Rikard Grankvist[1†], Arvin Chireh[1†], Mikael Sandell[1,3], Abdul Kadir Mukarram[4], Nasren Jaff[1], Ingrid Berggren[5], Hans Persson[6], Cecilia Linde[7,8], Fabian Arnberg[1,2], Johan Lundberg[1,2], Martin Ugander[9], Gioele La Manno[10], Stefan Jonsson[11], Carsten O. Daub[4], Staffan Holmin*[1,2]

† RG and AC contributed equally to this study.

Affiliations

1. Department of Clinical Neuroscience, Karolinska Institutet, Solna, Sweden.
2. Department of Neuroradiology, Karolinska University Hospital, Solna, Sweden.
3. Department of Micro and Nanosystems, Royal Institute of Technology, Stockholm, Sweden.
4. Department of Biosciences and Nutrition, Karolinska Institutet, Solna, Sweden.
5. Department of Molecular Medicine and Surgery, Karolinska Institutet, Solna, Sweden.
6. Department of Clinical Sciences, Danderyd Hospital, Karolinska Institutet, Stockholm, Sweden.
7. Department of Medicine, Karolinska Institutet, Solna, Sweden.
8. Heart and Vascular Theme, Karolinska University Hospital, Solna, Sweden.
9. Department of Clinical Physiology, Karolinska University Hospital, and Karolinska Institutet, Stockholm, Sweden. Charles Perkins Center, University of Sydney, Sydney, Australia. Kolling Institute, Royal North Shore Hospital, and Northern Clinical School, Sydney Medical School, University of Sydney, Sydney, Australia
10. Brain Mind Institute, School of Life Sciences, École Polytechnique Fédérale de Lausanne, Lausanne, Switzerland.
11. Department of Materials Science and Engineering, Royal Institute of Technology, Stockholm, Sweden.

Supplemental methods

Histology

Samples were placed directly on glass slides (Superfrost Plus, VWR, Radnor, PA, USA) and frozen. Slides were later air-dried, rehydrated with water and stained with either May-Grünwald-giemsa or hematoxylin-eosin (Histolab, Gothenburg, Sweden) according to manufacturer recommendations. Stained samples were mounted with DPX mountant (Sigma-Aldrich, St Louis, MO, USA), and imaged with brightfield microscopy using a Zeiss Axio Scope A1, and images where acquired using a microscope specific camera (AxioCam MRc5, Zeiss AB, Sweden).

Immunofluorescence

Samples were placed directly on glass slides (Superfrost Plus, VWR, Radnor, PA, USA) and frozen. Slides were later air-dried, rehydrated in phosphate-buffered saline (PBS, Sigma-Aldrich, St Louis, MO, USA), fixed in 4% buffered paraformaldehyde for 10 min at room temperature (RT), rinsed in PBS and blocked with normal goat serum 15 ml/ml in 1% bovine serum albumin and 0.3% Triton X-100 in PBS (blocking buffer, all reagents from Sigma-Aldrich, St Louis, MO, USA). The primary antibody for troponin I at dilution 1:200 in blocking buffer (mouse anti-swine, ab19615, Abcam, Cambridge, United Kingdom) was added for incubation overnight at +4°C. In negative controls, slides were incubated with blocking buffer only. Sections were washed three times in PBS and incubated Alexa488 donkey anti-mouse (Invitrogen, Carlsbad, Germany) for one hour at RT. After washing three times in PBS, the cell nuclei were stained with DAPI (Sigma, Sweden), prior to mounting the slides using a PBS:glycerol (1:3) mixture. Evaluation of staining was performed by fluorescence microscopy using a Zeiss Axio Scope A1 and images where acquired using a microscope specific camera, AxioCam MRc5 (Zeiss AB, Sweden).

Supplemental Figures

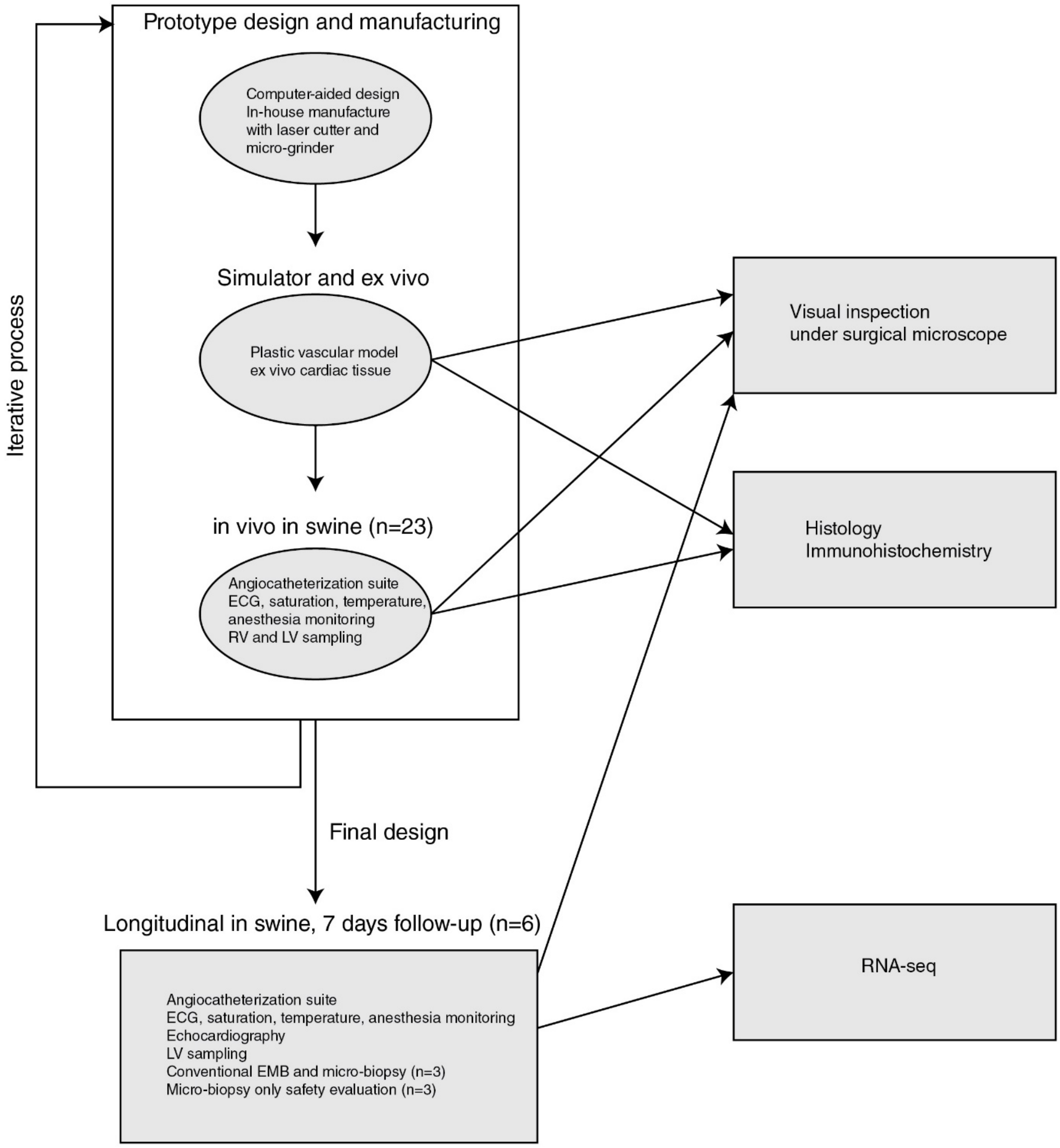

Figure S1. Experimental Design.

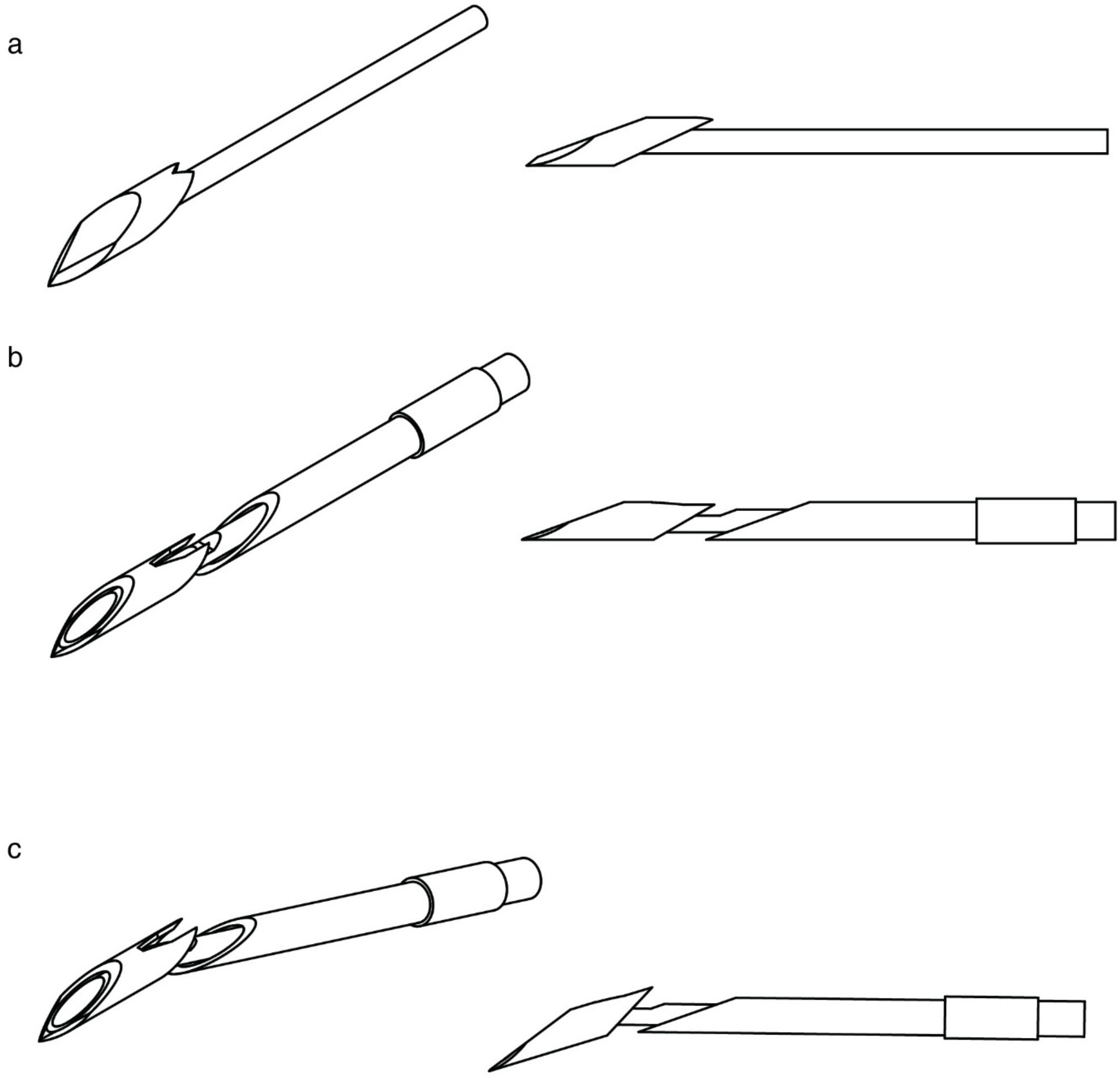

Figure S2. Select iterations of micro-EMB device design in chronological order. In design **(A)**, shown without the cutting tube, there is no sample collection notch, and the cutout in the proximal end of the cutting head is smaller. In design **(B)**, the notch has been added and the cutout is larger, but the cutting head is not angled in relation to the axis of the tube and wire. In design **(C)**, the cutting head is angled, but the cutting tube is sharpened at an angle similar to the cutting head, later changed to the circumferential cutting tube (Figure 1A in the main article).

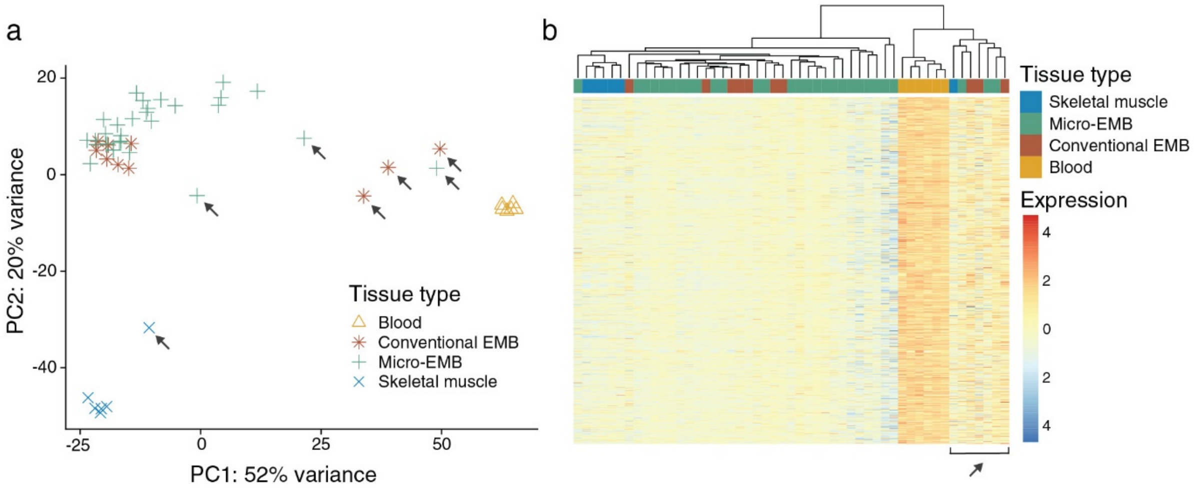

Figure S3 Identification of blood contamination. **(A)** PCA plot of gene expression before exclusion of blood contaminated samples (n = 51). Seven samples (black arrows) deviate from their reference groups. **(B)** Gene expression heatmap for blood specific genes (n = 604). Expression values are scaled per row. The dendrogram shows hierarchical clustering based on sample-to-sample distances. The clustering algorithm separates the samples into two main groups, where a subset (indicated by brackets and arrow) is grouped together with blood samples. The same samples are marked by arrows in subfigure **(A)**.
